# Synthesis of nonperipherally tetra-[5-(diethylamino)-2-formylphenoxy] substituted metallophthalocyanines and their electrochemistry

**DOI:** 10.3906/kim-2007-47

**Published:** 2021-02-17

**Authors:** Turgut KELEŞ, Dilek ÜNLÜER, Zekeriya BIYIKLIOĞLU*, Yasemin ÜNVER

**Affiliations:** 1 Central Research Laboratory Application and Research Center, Recep Tayyip Erdoğan University, Rize Turkey; 2 Karadeniz Technical University, Department of Chemistry, Trabzon Turkey

**Keywords:** Synthesis, diethylamino, phthalocyanine, electrochemistry

## Abstract

3-[5-(diethylamino)-2-formylphenoxy]phthalonitrile ( **n-TY-CN** ), metallophthalocyanines **n-TY-Co** , **n-TY-Cu** , and **n-TY-Mn** bearing [5-(diethylamino)-2-formylphenoxy] groups at nonperipheral positions were prepared for the first time. These compounds were characterized with IR, NMR (only for **n-TY-CN** ), mass and UV-vis (except **n-TY-CN** ) spectroscopy. Voltammetric characterizations of **n-TY-Co** , **n-TY-Cu** , and **n-TY-Mn** revealed that while **n-TY-Co** , **n-TY-Cu** , and **n-TY-Mn** showed characteristic Pc ring and/or metal-based reduction reaction, **n-TY-Co** , **n-TY-Cu** , and **n-TY-Mn** were coated on the working electrode during the oxidation processes owing to the cationic electropolymerizations of the [5-(diethylamino)-2-formylphenoxy] substituents.

## 1. Introduction

Phthalocyanines are all-purpose and durable compounds [1]. They have an extended π-conjugation system and different types of central metals [2]. In addition, their unique electronic and chemical properties, physical and optical properties, as well as chemical flexibility allow for the preparation of a variety of related structures and diverse applications ranging from industrial to biological areas [3,4]. Phthalocyanines have been used in different areas such as photodynamic therapy [5,6], nonlinear optical materials [7], electrochemical sensors [8,9], biosensors [10], solar cells [11,12], light-emitting devices [13], liquid crystals [14], electrocatalysts [15], and electropolymerization [16,17]. The solubility of unsubstituted phthalocyanines is very low in organic solvents. This condition affects the use of phthalocyanines in many areas. The solubility of phthalocyanines can be increased with substitution of phenoxy, alkyl, alkoxy, long chain, and bulky groups at peripheral, nonperipheral, or axial positions of the phthalocyanine [18–20]. Diethylamino groups can be used in many applications such as anticancer agents [21], DNA interactions [22], and photodynamic therapy [23]. In addition, the electrochemical features of diethylamino groups are significant. In the literature, it is shown that the introduction of diethylamino groups into the peripheral/nonperipheral positions of phthalocyanines increased their electrochemical and electropolymerization properties [24–27]. Here, we wondered how the presence of phthalocyanines in the nonperipheral positions of the diethylamino group affects the electrochemical properties of phthalocyanine compounds. For this reason, in this work, we combined these two functional compounds (diethylamino and phthalocyanine) into a single compound. In this study, we have synthesized nonperipherally tetra-[5-(diethylamino)-2-formylphenoxy] substituted **n-TY-Co** , **n-TY-Cu** , and **n-TY-Mn** , and investigated their electrochemical properties. 

## 2. Experimental details

Information about equipment, materials, and electrochemistry experiments is provided in the Supplementary Information section.

### 2.1. Synthesis

#### 2.1.1. 3-[5-(diethylamino)-2-formylphenoxy]phthalonitrile (n-TY-CN)

4-(diethylamino)-2-hydroxybenzaldehyde (1 g, 5.2 mmol), 3-nitrophthalonitrile (0.9 g, 5.2 mmol), and dry K2CO3 (2.15 g, 15.6 mmol) were dissolved in DMF (15 mL) at 55 °C under nitrogen atmosphere for 96 h. Then, the solution was estranged and spilled into iced water (200 mL). **n-TY-CN** was crystallized from ethanol and purified using column chromatography on aluminum oxide with chloroform. Yield: 0.47 g (33%), m.p. 155–157 °C. IR (ATR), ν/cm^-1^: 3097 (Ar–H), 2974–2877 (Aliph. C–H), 2225 (C≡N), 1674 (C=O), 1603, 1543, 1445, 1377, 1260, 1192, 1094, 1074, 844, 799, 689. ^1^H NMR (400 MHz, DMSO-d_*6*_), (δ): 9.71 (s, 1H, =CH), 7.81–7.73 (m, 3H, Ar–H), 7.21 (d, 1H, Ar–H), 6.78 (d, 1H, Ar–H), 6.52 (s, 1H, Ar–H), 3.46–3.41 (m, 4H, CH2–N), 1.10 (t, 6H, CH_3_). ^13^C-NMR (100 MHz, DMSO- *d*_*6*_), (δ): 185.97, 160.97, 156.84, 154.05, 136.34, 133.73, 128.13, 121.15, 116.17, 116.07, 115.46, 113.94, 109.59, 104.91, 103.36, 44.67, 12.73. MS (ESI), (m/z) calcl. 319; found: 342.03 [M + Na]^+^.

#### 2.1.2. 1(4),8(11),15(18),22(25)-tetrakis-[5-(diethylamino)-2-formylphenoxy]-phthalocyaninato cobalt (II) (n-TY-Co)

3-[5-(diethylamino)-2-formylphenoxy]phthalonitrile (150 mg, 0.36 mmol), CoCl_2_ (24 mg, 0.18 mmol), 1-pentanol (2.5 mL), and DBU (3 drops) were stirred at 160 °C for 24 h under N_2_ atmosphere. The product was precipitated with hexane. **n-TY-Co** was obtained by column chromatography using basic aluminum oxide and CHCl3 as a solvent. Yield: 27 mg (17%), m.p. > 300 °C. IR (ATR), ν/cm^-1^: 3066 (Ar–H), 2959–2852 (Aliph. C–H), 1667 (C=O), 1590, 1517, 1400, 1353, 1238, 1196, 1136, 1090, 985, 797, 746, 692. UV-vis (THF) λmax nm (log e): 678 (5.02), 621 (4.48), 311 (5.06). MALDI-TOF-MS (DIT) m/z: 1336.04 [M]^+^, 1508.00 [M + DIT–C_4_H_6_]^+^, 1612.48 [M + DIT + 3H_2_O–4H]^+^, 1697.22 [M + DIT + 3K + H_2_O]^+^.

#### 2.1.3. 1(4),8(11),15(18),22(25)-tetrakis-[5-(diethylamino)-2-formylphenoxy]-phthalocyaninato copper (II) (n-TY-Cu)

**n-TY-Cu** was synthesized similarly to **n-TY-Co** by using CuCl_2_ instead of CoCl_2_. Yield: 40 mg (38%), m.p. > 300 °C. IR (ATR), ν/cm^-1^: 3065–3035 (Ar–H), 2967–2867 (Aliph. C–H), 1664 (C=O), 1590, 1519, 1480, 1397, 1330, 1235, 1194, 1124, 1078, 890, 799, 742, 630. UV-vis (THF) λmax nm (log e): 697 (5.03), 662 (4.36), 629 (4.35), 340 (4.87). MALDI-TOF-MS (CHCA) m/z: 1250.11 [M–C_6_H_17_]^+^, 1319.48 [M–CH_8_]^+^, 1339.70 [M]^+^, 1402.70 [M + Na + K + H]^+^, 1425.63 [M + 2Na + K + H]^+^, 1511.27 [M + CHCA–H_2_O + H]^+^.

#### 2.1.4. 1(4),8(11),15(18),22(25)-tetrakis-[5-(diethylamino)-2-formylphenoxy]-phthalocyaninato manganese (III) chloride (n-TY-Mn)

**n-TY-Mn** was synthesized similarly to **n-TY-Co** by using MnCl_2_ instead of CoCl_2_. Yield: 40 mg (37%), m.p. > 300 °C. IR (ATR), ν/cm^-1^: 3060 (Ar–H), 2965–2856 (Aliph. C–H), 1663 (C=O), 1594, 1518, 1482, 1326, 1239, 1093, 1067, 1013, 894, 797, 741, 692. UV-vis (THF) λmax nm (log e): 795 (4.78), 755 (4.95), 527 (4.36), 345 (5.20). MALDI-TOF-MS (DIT) m/z: 1308.54 [M–C_4_H_11_]^+^, 1331.85 [M–Cl–H]^+^, 1348.80 [M–CH_7_]^+^, 1417.65 [M + 2Na + K–Cl]^+^.

## 3. Results and discussion

### 3.1. Synthesis and characterization

**Figure 1:**
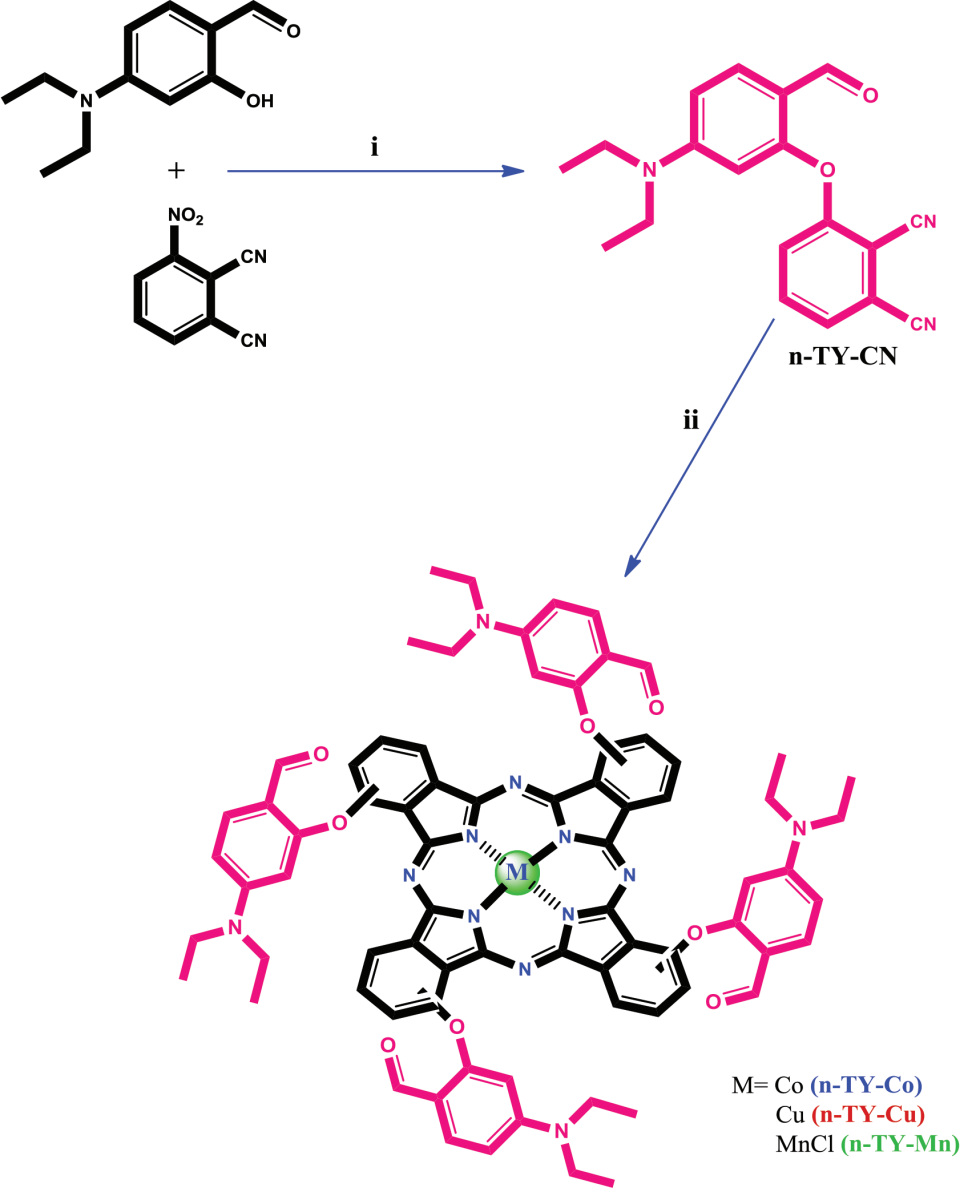
The synthesis of n-TY-Co, n-TY-Cu, and n-TY-Mn containing [5-(diethylamino)-2-formylphenoxy] groups. (i) DMF, K_2_CO_3_, 55 °C; (ii) n-pentanol, DBU, metal salts, 160 °C.

**Figure 2:**
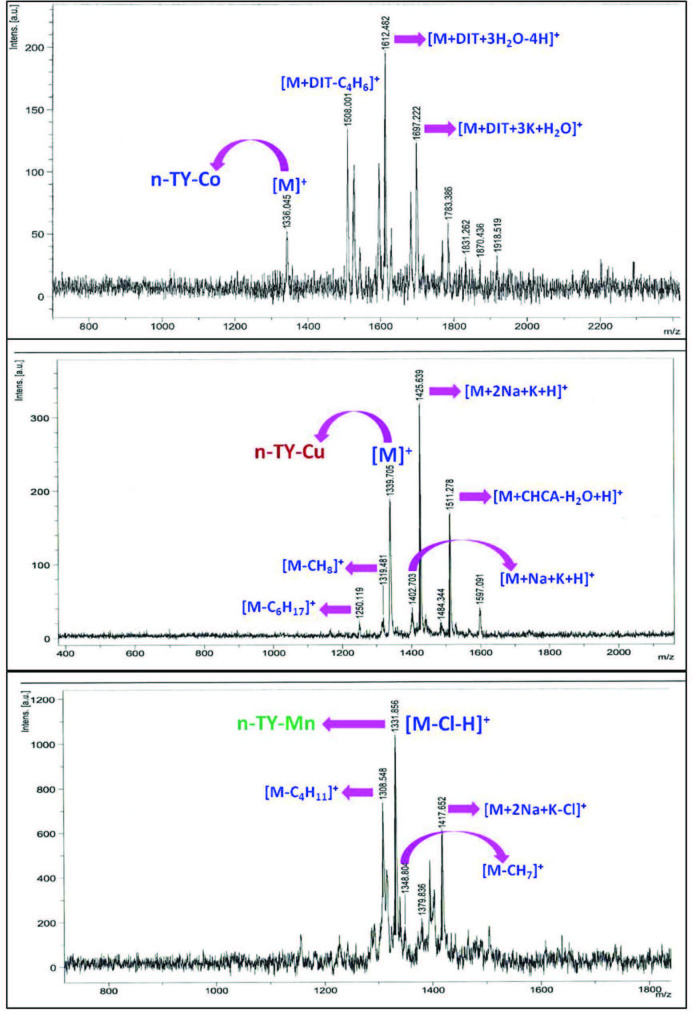
MALDI-TOF MS spectrum of n-TY-Co, n-TY-Cu, and n-TY-Mn.

**Figure 3:**
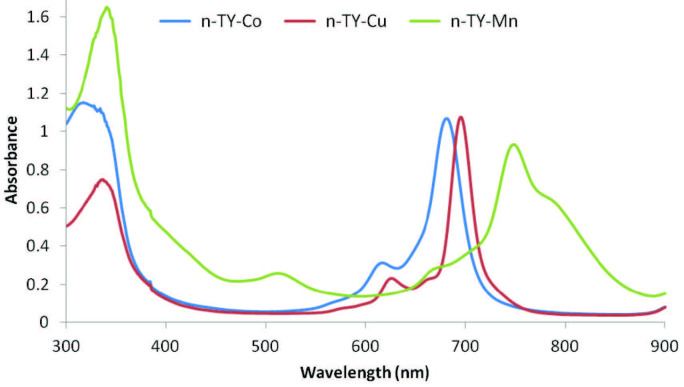
UV-vis spectra of n-TY-Co, n-TY-Cu, and n-TY-Mn in THF.

In this study, 3-[5-(diethylamino)-2-formylphenoxy]phthalonitrile ( **n-TY-CN** ), nonperipherally tetra-[5-(diethylamino)-2-formylphenoxy] substituted metallophthalocyanines **(n-TY-Co** , **n-TY-Cu** , **n-TY-Mn)** were synthesized for the first time and characterized with IR, NMR (only for **n-TY-CN** ), mass and UV-vis (except **n-TY-CN** ) spectroscopy. The synthesis of **n-TY-Co** , **n-TY-Cu** , and **n-TY-Mn** is shown in Figure 1. The detailed synthesis of 3-[5-(diethylamino)-2-formylphenoxy]phthalonitrile ( **n-TY-CN** ) and metallophthalocyanines ( **n-TY-Co** , **n-TY-Cu** , **n-TY-Mn** ) is given in Supplementary Information. Stretching vibrations of C=N groups at 2225 cm^-1^ were observed in the IR spectrum of **n-TY-CN** . In 1H-NMR spectrum of **n-TY-CN** , aldehyde and aromatic protons were detected at 9.71 and 7.81–6.52 ppm. Also, the aliphatic CH_2-_N and –CH_3_ protons were observed at 3.46–3.41 ppm as a multiplet, 1.10 ppm as a triplet. The aromatic and aliphatic carbon signals were observed at 185.97–103.36 and 44.67–12.73 ppm. In the IR spectra of **n-TY-Co** , **n-TY-Cu** , and **n-TY-Mn** , the sharp CºN stretching vibration disappeared. Also, C=O stretching vibrations of **n-TY-Co** , **n-TY-Cu** , and **n-TY-Mn** were shown at 1667, 1664, and 1663 cm^-1^, respectively. NMR spectra of **n-TY-Co** , **n-TY-Cu** , and **n-TY-Mn** could not be obtained owing to paramagnetic Co(II), Cu(II), Mn(III) ions [28]. MALDI-TOF mass spectra were obtained in dithranol (DIT) for **n-TY-Co, n-TY-Mn** , and alpha-cyano4-hydroxycinnamic acid (CHCA) for **n-TY-Cu** as MALDI matrix materials. The molecular ion peaks of **n-TY-CN,** **n-TY-Co** , **n-TY-Cu** , and **n-TY-Mn** were observed as 342.03 [M + Na]^+^, 1336.04 [M]^+^, 1339.70 [M]^+^, and 1331.85 [M–Cl–H]+, respectively (Figure 2). In the UV-vis spectra of **n-TY-Co** , **n-TY-Cu** , and **n-TY-Mn** in THF, the characteristic Q band (678 nm for **n-TY-Co** , 697 nm for **n-TY-Cu** , 795 nm for **n-TY-Mn** ) and B band (311 nm for **n-TY-Co** , 340 nm for **n-TY-Cu** , 345 nm for **n-TY-Mn** ) were observed (Figure 3). As shown in Figure 3, **n-TY-Mn** showed aggregation in THF, but **n-TY-Co** and **n-TY-Cu** did not show any aggregation in THF.

### 3.2. Voltammetric studies

**Figure 4:**
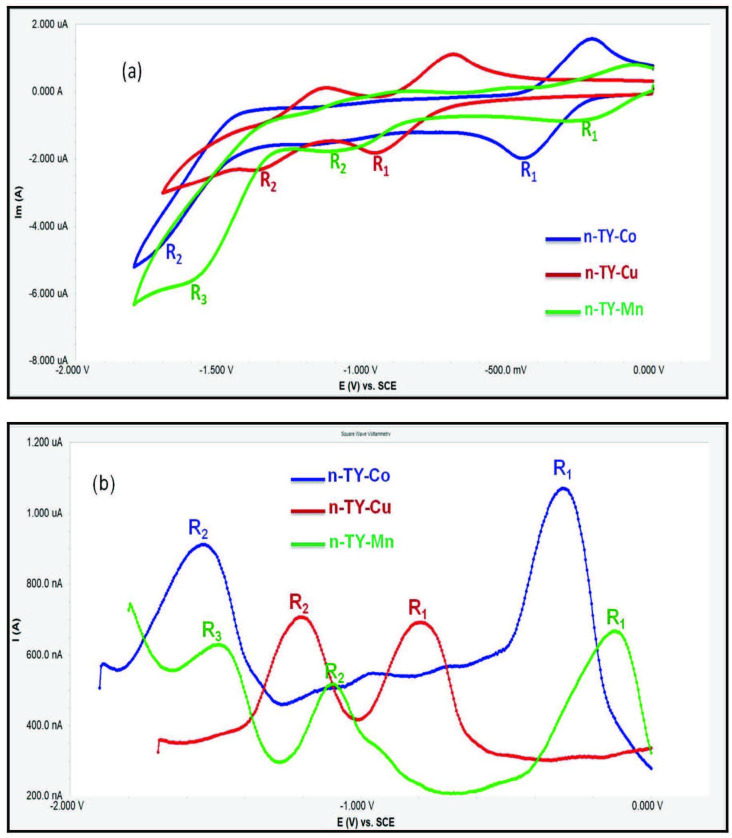
(a) CV graph of n-TY-Co, n-TY-Cu, and n-TY-Mn in TBAP/DCM electrolyte system on platin working electrode. (b) SWV graph of n-TY-Co, n-TY-Cu, and n-TY-Mn in TBAP/DCM electrolyte system on platin working electrode.

**Figure 5:**
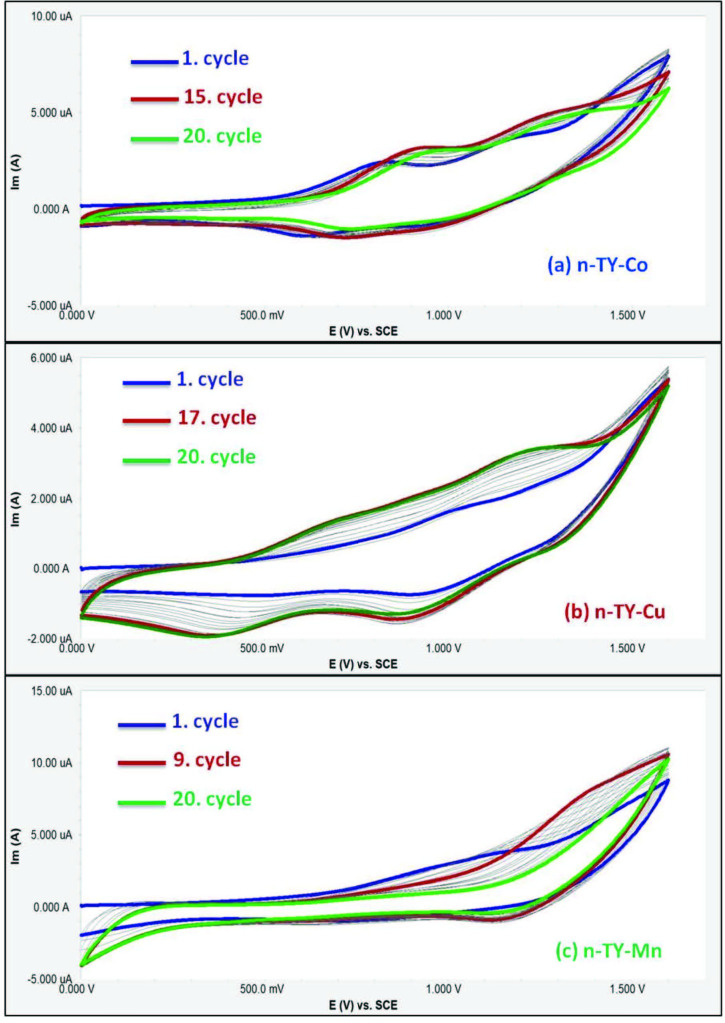
(a) Repetitive CVs of n-TY-Co in TBAP/DCM electrolyte system on platin working electrode at 0.100 mV s^-1^ scan rate. (b) Repetitive CVs of n-TY-Cu in TBAP/DCM electrolyte system on platin working electrode at 0.100 mV s–1 scan rate. (c) Repetitive CVs of n-TY-Mn in TBAP/DCM electrolyte system on platin working electrode at 0.100 mV s^-1^ scan rate.

Voltammetric analysis of **n-TY-Co** , **n-TY-Cu** , and **n-TY-Mn** was achieved in DCM using a (DCM)/(TBAP) electrolyte system on a Pt working electrode. The voltammetric data is shown in Table. Figures 4a and 4b show the CV and SWV responses of **n-TY-Co** , **n-TY-Cu** , and **n-TY-Mn** . As shown in Figure 4, **n-TY-Co** showed metal-based reversible and Pc-based quasireversible reduction reactions labeled as R_1_ (E_1/2_ = –0.33 V), R_2_ (E_1/2_ = –1.53 V). Also, **n-TY-Cu** gave two reversible, Pc-based reduction reactions (E_1/2_ = –0.82 V, –1.25 V). On the other hand, **n-TY-Mn** illustrated reversible (R_1_ E_1/2_ = –0.13 V) and quasireversible (R_2_ E_1/2_ = –0.97 V, R_3_ E_1/2_ = –1.46 V) reduction processes, which were assigned to [Cl–Mn^III^Pc^-2^] / [Cl–Mn^II^Pc^-2^]^-1^, [Mn^II^Pc^-2^] / [MnIPc^-2^]^-1^, [MnIPc^-2^]–1 / [Mn^I^Pc–3]^-2^ couples. The ligands containing diethylamino groups polymerize during the oxidation reaction [29,30]. For this reason, **n-TY-Co** , **n-TY-Cu** , and **n-TY-Mn** containing [5-(diethylamino)-2-formylphenoxy] groups were electropolymerized during the oxidation reaction. Figure 5 shows the CV responses of **n-TY-Co** , **n-TY-Cu** , and **n-TY-Mn** during repetitive CV cycles. Figure 5a shows the repetitive CV responses of the **n-TY-Co** recorded at 0.100 V s^-1^ scan rate within a positive potential window of DCM/TBAP. During the first anodic CV cycle, **n-TY-Co** shows two oxidation peaks at 0.81 and 1.18 V. During the second to fifteenth CV cycles, the anodic waves increase with potential shifts at 0.92 and 1.27 V. After this point, the potentials decrease with the ultimate disappearance of current intensity after the 20th cycle. Figure 5b illustrates CV responses of **n-TY-Cu** during repetitive CV cycles. During the first anodic scan, an anodic wave at 1.05 V and its reverse cathodic couple is recorded at 0.92 V. During the second to seventeenth CV cycles, the anodic waves increase with a potential shift at 1.20 V. Also, during the consecutive third CV cycle, two new cathodic waves are recorded at 0.48 and 0.89 V. These new waves increase in current intensity with a negative potential shift to 0.33 and 0.84 V during the 20th CV cycle. Figure 5c shows the repetitive CV cycles **n-TY-Mn** . During the first CV cycle, an oxidation peak at 1.10 V is observed. During the second to ninth CV cycles, this peak increases in current intensity with a positive potential shift. After this point, the potentials decrease with the ultimate disappearance of current intensity after the 20th cycle. These voltammetric responses show the electropolymerization of the **n-TY-Co** , **n-TY-Cu** , and **n-TY-Mn** on the working electrode due to the oxidation of diethylamino groups of the metallophthalocyanines. In order to prepare a composite electrode, electropolymerization is required [31]. Also, electropolymerization necessitates electroactive groups, for example, morpholine [32], diethylamino [33], and thiophene [34] in the monomer system. For these reasons, the electropolymerization feature may allow the synthesized phthalocyanines ( **n-TY-Co** , **n-TY-Cu** , **n-TY-Mn** ) to be used in different electrochemical areas, for example as electrocatalyts, electrochromic materials, and electrochemical sensors [35]. 

**Table:** Electrochemical results of the n-TY-Co, n-TY-Cu, n-TY-Mn. All data were given versus SCE.

Pcs		Oxidation of substituent		R_1_	R_2_	R_3_
n-TY-Co	^a^E_1/2_	0.81	1.18	–0.33	–1.53	-
n-TY-Cu	^a^E_1/2_	1.05	-	–0.82	1.25	-
n-TY-Mn	^a^E_1/2_	1.10	-	–0.13	–0.97	–1.46

^a^: E_1/2_ values ((E_pa_+E_pc_)/2) were given versus SCE at 0.100 V s^-1^ scan rate.

## 4. Conclusion

In this work, nonperipherally tetra-[5-(diethylamino)-2-formylphenoxy] substituted **n-TY-Co** , **n-TY-Cu** , and **n-TY-Mn** were synthesized and characterized. Voltammetric analysis of **n-TY-Co** , **n-TY-Cu** , **n-TY-Mn** was defined by using cyclic and square wave voltammetry. Voltammetric results show that **n-TY-Co** and **n-TY-Cu** give two reduction processes, but **n-TY-Mn** gives three reduction processes during the cathodic scans. Also, **n-TY-Co** , **n-TY-Cu** , and **n-TY-Mn** revealed electropolymerization responses during the anodic scans because the ligands containing [5-(diethylamino)-2-formylphenoxy] groups polymerize during the oxidation reaction.

Supplementary Materials
