# Supplementary material for: Synthesis of nonperipherally tetra-[5-(diethylamino)-2-formylphenoxy] substituted metallophthalocyanines and their electrochemistry
Source: Turk J Chem. 2021 Feb 17;45(1):17–25. doi: 10.3906/kim-2007-47 (PMC7925299; doi:10.3906/kim-2007-47)
Supplement: Supplementary file 1 — Supplementary Materials [file turkjchem-45-17-sup001.pdf]

SUPPLEMENTARY INFORMATION

1. Materials and equipment

4-(diethylamino)-2-hydroxybenzaldehyde and 3-nitrophthalonitrile were purchased from commercial suppliers. All reagents and solvents were of reagent grade quality and were obtained from commercial suppliers. The IR spectra were recorded on a Perkin Elmer 1600 FT-IR spectrophotometer using KBr pellets. ^{1}H and ^{13}C-NMR spectra were recorded on a Bruker Avance III 400 MHz spectrometers in DMSO-d_6 and chemical shifts were reported (δ) relative to Me_4Si as an internal standard. MALDI-MS of complexes were obtained in dithranol (DIT), α-cyano-4-hydroxycinnamic acid (CHCA) as MALDI matrices using nitrogen laser accumulating 50 laser shots using Bruker Microflex LT MALDI-TOF mass spectrometer (Bremen, Germany). Optical spectra in the UV-vis region were recorded with a Perkin Elmer Lambda 25 spectrophotometer.

2. Electrochemical measurements

The cyclic voltammetry (CV) and square wave voltammetry (SWV) measurements were carried out with Gamry Interface 1000 potentiostat/galvanostat controlled by an external Pc and utilizing a three-electrode configuration at 25 °C. The working electrode was a Pt disc with a surface area of 0.071 cm^2. A Pt wire served as the counter electrode. Saturated calomel electrode (SCE) was employed as the reference electrode and separated from the bulk of the solution by a double bridge. Electrochemical grade TBAP in extra pure DCM was employed as the supporting electrolyte at a concentration of 0.10 mol dm^{-3}.

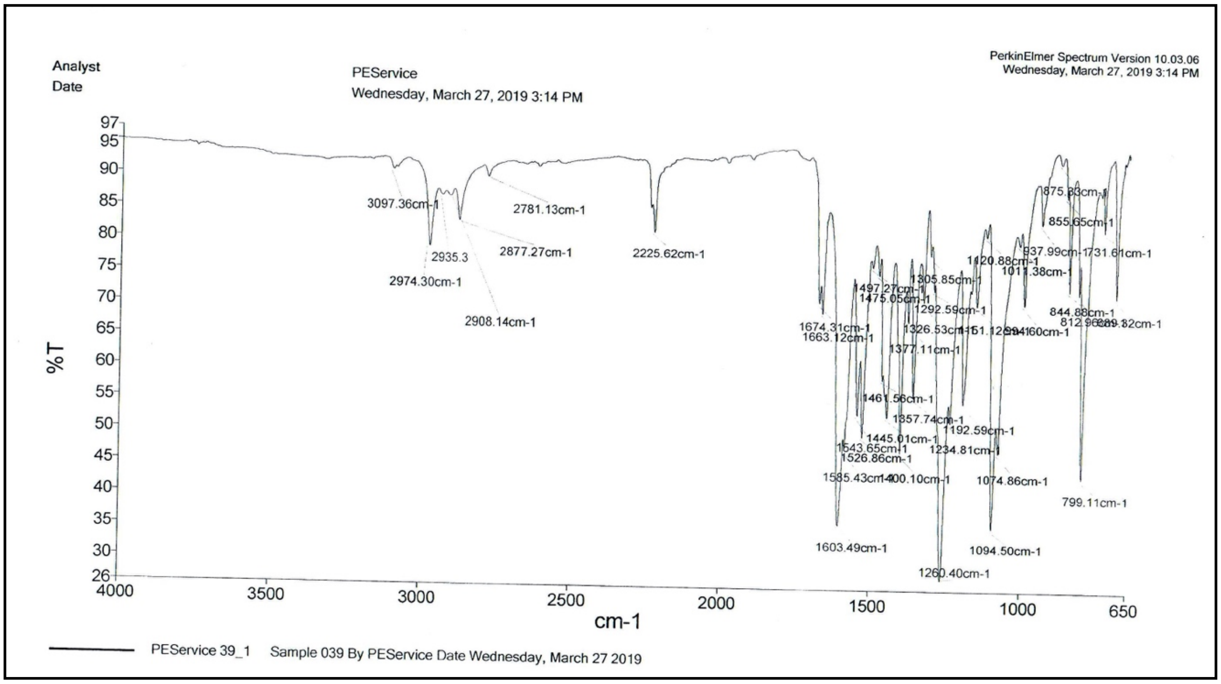

Figure S1. IR spectra of **n-TY-CN**.

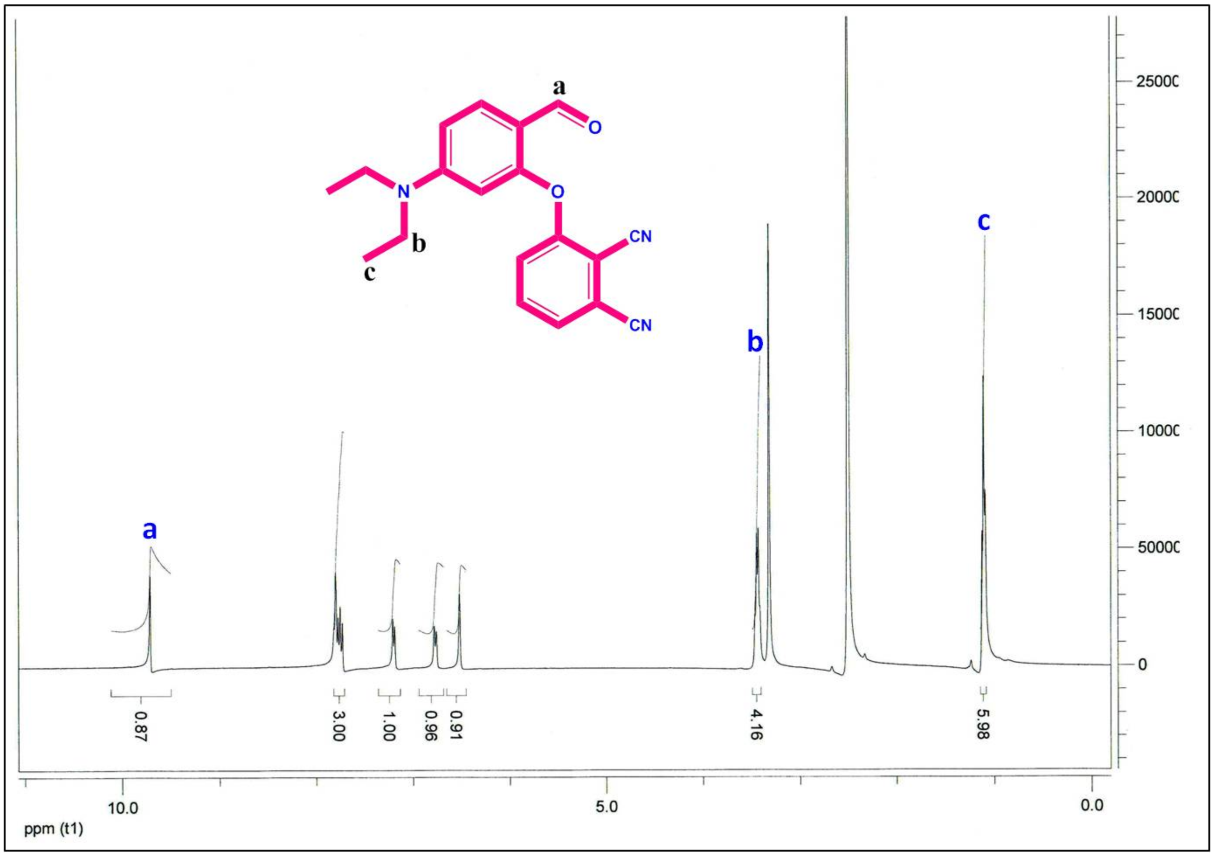

Figure S2. ^{1}H-NMR spectrum of **n-TY-CN** in DMSO-d_6.

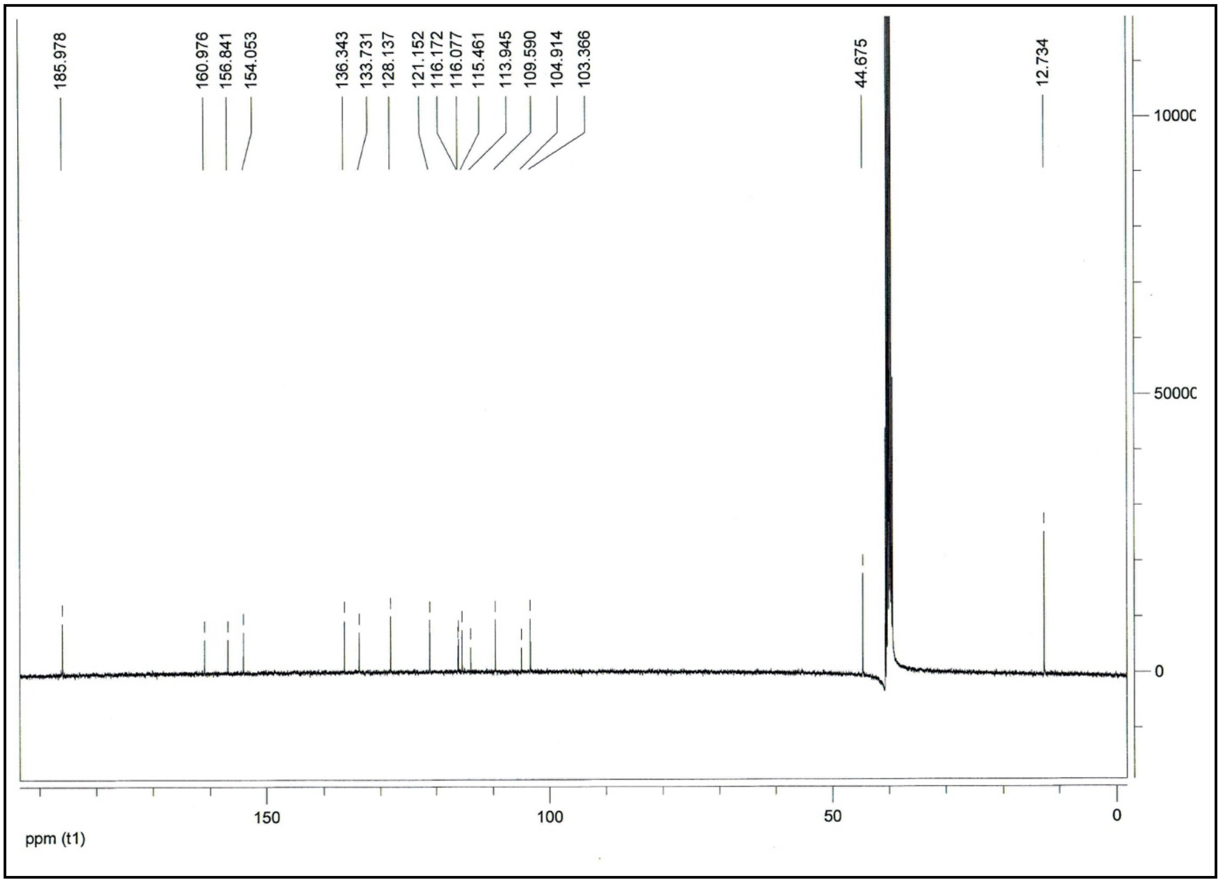

Figure S3. ^{13}C-NMR spectrum of **n-TY-CN** in DMSO-d_6.

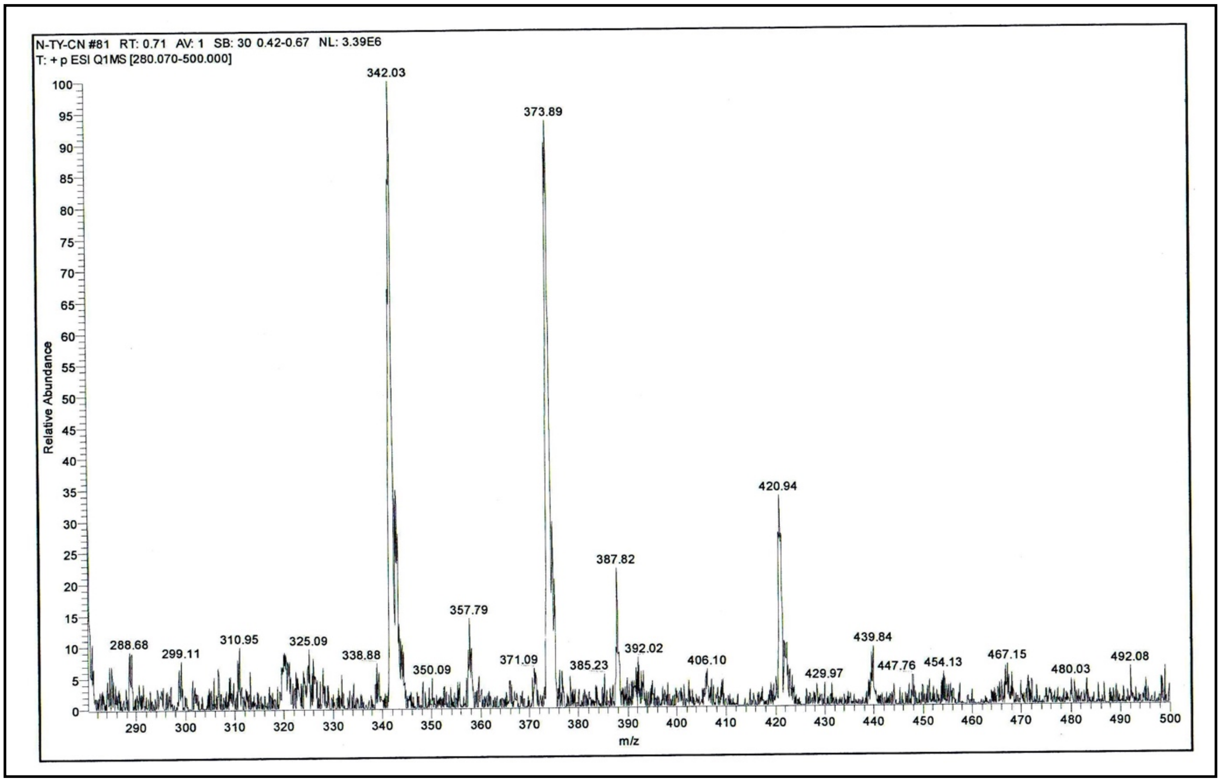

Figure S4. Mass of **n-TY-CN**.

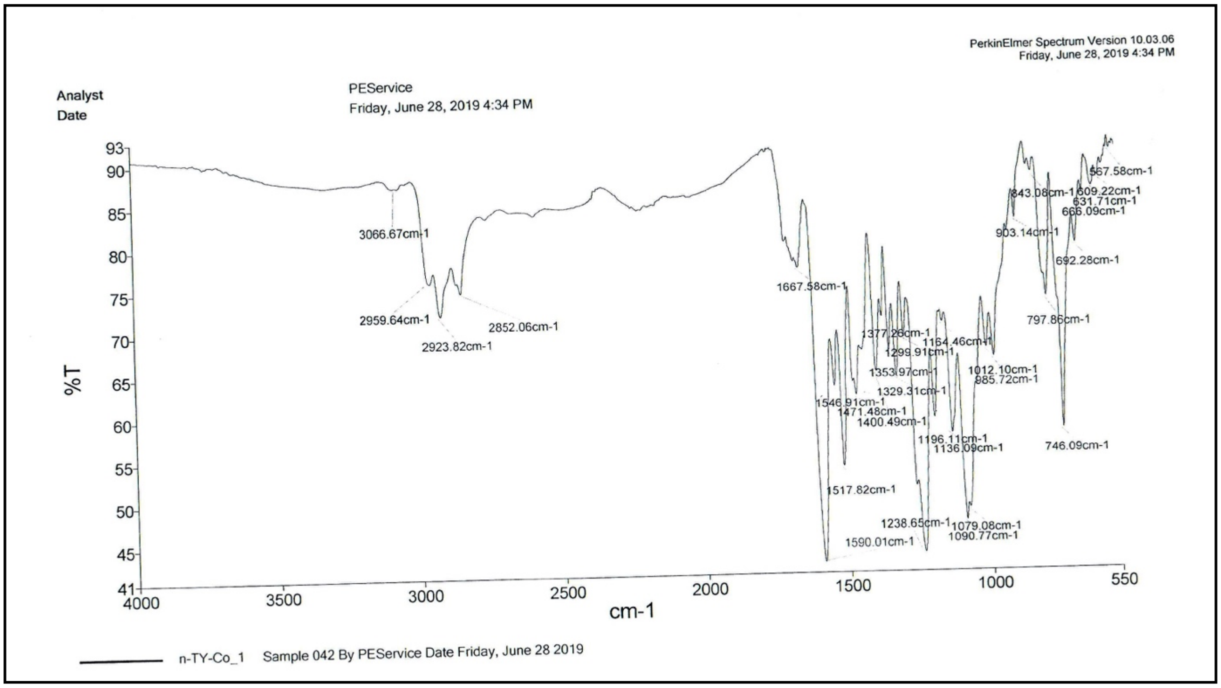

Figure S5. IR spectra of **n-TY-Co**.

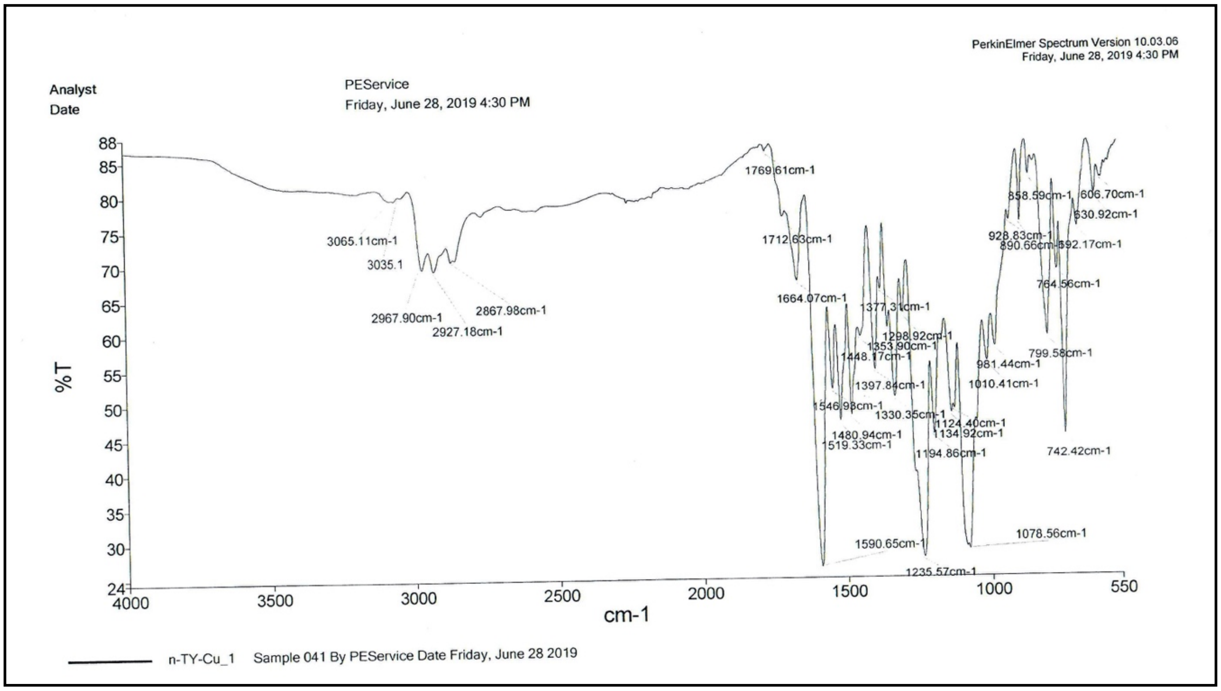

Figure S6. IR spectra of **n-TY-Cu**.

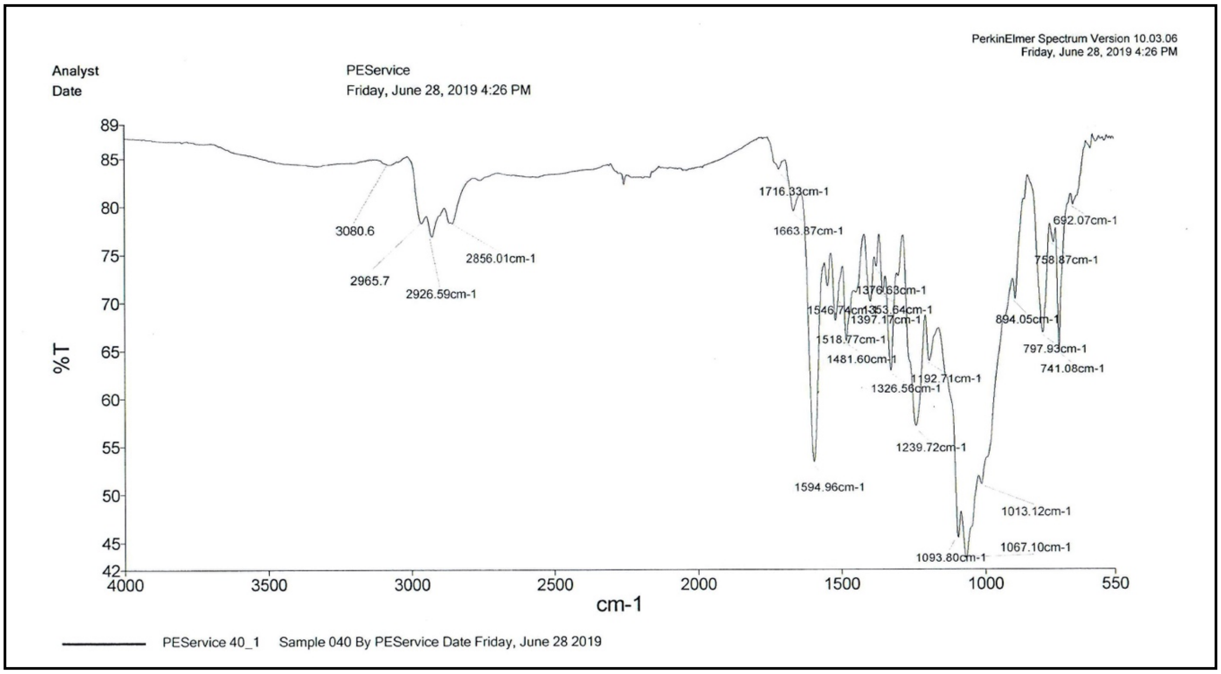

Figure S7. IR spectra of **n-TY-Mn**.
